# Molecular mechanism by which the Notch signaling pathway regulates autophagy in a rat model of pulmonary fibrosis in pigeon breeder’s lung

**DOI:** 10.1515/med-2023-0629

**Published:** 2023-02-08

**Authors:** Yafang Li, Zhichuang Lian, Qifeng Li, Wei Ding, Wenyi Wang, Ling Zhang, Xirennayi Muhataer, Yuan Zhou, Xiaohong Yang, Chao Wu

**Affiliations:** Department of Respiratory and Critical Care Medicine, People’s Hospital of Xinjiang Uygur Autonomous Region, 830001 Urumqi, China; Xinjiang Clinical Research Center for Interstitial Lung Diseases, 830001 Urumqi, China

**Keywords:** hypersensitivity pneumonitis, pigeon breeder’s lung, pulmonary fibrosis, Notch signaling pathway, autophagy

## Abstract

This study investigated the molecular mechanisms underlying the involvement of the Notch signaling pathway and autophagy in the development of pulmonary fibrosis in pigeon breeder’s lung (PBL). Rats were divided into control (Ctrl), PBL model (M), M + D (Notch signaling inhibition), M + W (autophagy inhibition), and M + R (autophagy induction) groups. Lyophilized protein powder from pigeon shedding materials was used as an allergen to construct a fibrotic PBL rat model. The mechanism by which Notch signaling regulated autophagy in the pulmonary fibrosis of PBL was investigated by inhibiting the Notch pathway and interfering with autophagy. Pulmonary interstitial fibrosis was significantly greater in the M group and the M + W group than in the M + D and M + R groups. The expression of α-smooth muscle actin was significantly higher in the M, M + D, and M + W groups than in the Ctrl group (*P* < 0.05). The expression of the cell autophagy markers Beclin1 and LC3 was lower in the M, M + D, and M + W groups than in the Ctrl group (*P* < 0.05), whereas Beclin1 and LC3 expressions were higher in the M + D and M + R groups than in the M group. The levels of reactive oxygen species in serum and lung tissues were higher in the M, M + D, M + W, and M + R groups than in the Ctrl group (*P* < 0.05). The Notch signaling pathway is involved in the pathological process of pulmonary fibrosis in the rat model of PBL by regulating autophagy.

## 1 Introduction

Pigeon breeder’s lung (PBL) [1] is a kind of hypersensitivity pneumonitis (HP), which is a pulmonary inflammatory response induced by repeated inhalation of pigeon fur and other shedding materials by sensitive individuals. As the disease progresses, inflammatory injuries such as granuloma and pulmonary fibrosis occur successively. The mechanism of the evolution of its inflammatory damage remains unclear [2,3], and pulmonary fibrosis in some patients is still progressing after they are free of allergens. Therefore, it is necessary to study the mechanism of fibrosis of PBL.

The pathogenesis of pulmonary fibrosis is complex, and a variety of factors, such as cells, cytokines, and cell signaling pathways, are involved [4]. It is closely related to Notch signaling pathway abnormalities, autophagy, and oxidative stress [5–7]. The Notch pathway regulates processes including cell differentiation, proliferation, and autophagy. Notch can induce epithelial–mesenchymal transition (EMT) in alveolar epithelial cells through transforming growth factor β1 (TGF-β1), thereby causing pulmonary interstitial fibrosis, of which α-smooth muscle actin (α-SMA) is a marker [8]. Activation of Notch signaling can aggravate renal fibrosis by aggravating oxidative stress [9]. Notch can participate in the process of organ fibrosis through various mechanisms. Studies have also found the accumulation of reactive oxygen species (ROS) in fibroblasts of patients with pulmonary fibrosis, and their mitochondrial autophagy is attenuated [10,11]. Cellular autophagy is a homeostatic state, and either excessive or insufficient autophagy can damage cells. Beclin1 and LC3 are two signature proteins that represent the cellular autophagy level.

When the body produces excessive ROS or has too little antioxidant capacity, ROS will accumulate in tissues or cells, which can induce oxidative stress, resulting in lung tissue damage and remodeling [12]. The level of oxidative stress is increased in patients with idiopathic pulmonary fibrosis, and oxidative stress can induce autophagy in the process of pulmonary fibrosis; at the same time, ROS can damage mitochondria and be cleared through the autophagy pathway [13]. The mechanisms underlying the involvement of autophagy in pulmonary fibrosis may include inhibition of ROS production, proliferation and differentiation of fibroblasts, apoptosis of epithelial cells, and EMT [6,14]. Insufficient autophagy can accelerate the differentiation of fibroblasts into myofibroblasts, promote the expression of α-SMA, and lead to increased production of extracellular matrix, thereby accelerating the progression of pulmonary fibrosis. In summary, abnormalities in the Notch signaling pathway, dysfunction of autophagy, or inactivated autophagy play an important role in pulmonary fibrotic diseases. However, the possible interaction mechanism between the Notch signaling pathway, autophagy, and oxidative stress remains unclear.

We used pigeon feathers and other shedding materials to make lyophilized allergen powder and successfully constructed an HP-fibrotic PBL rat model [15]. After interfering with Notch signaling and autophagy, we observed lung tissue pathology, α-SMA expression, autophagy protein, and oxidative stress marker expression and preliminarily elucidated the mechanisms by which Notch signaling, autophagy, and oxidative stress regulate the formation of pulmonary fibrosis of PBL. This study provides a novel target for blocking the inflammatory injury of HP.

## 2 Materials and methods

### 2.1 Experimental animals

In this study, clean-grade, healthy, adult Sprague–Dawley rats, both male and female, aged 6–8 weeks, weighing 180–200 g, were purchased from Shanghai Xipuer-Bikai Laboratory Animal Co., Ltd, with license number SCXK (Shanghai) 2018-0006. All operations and experimental procedures were in accordance with the Regulations on the Management of Laboratory Animals.

This experiment was approved by the Ethics Committee of our hospital, with approval number KY201803715 of the Ethics Committee of Xinjiang District Hospital. All methods were carried out in accordance with the relevant guidelines and regulations and with the ARRIVE guidelines.

### 2.2 Experimental methods

#### 2.2.1 Extraction of allergens from pigeon shedding materials

The collected fresh pigeon feathers and dander were dissolved in phosphate-buffered saline (PBS, pH 7.4) at a concentration of 1:20 and thoroughly stirred for 24 h. Lyophilized protein powder from pigeon shedding materials of feathers and dander were prepared after filtering, removing water, and other operations and stored at 1–4°C for future use. The prepared lyophilized powder contained a large amount of active allergens.

#### 2.2.2 Animal experiments

We have established a rat model of PBL at different pathological stages in earlier studies. In those studies and here, the allergenic lyophilized powder suspension was administered by airway instillation at 300 µL/20 µg once a week as an appropriate way to induce fibrosis in rats. Model establishment and drug administration were performed according to the following groups (five groups, nine animals in each group): (1) normal control group (Ctrl): airway instillation of normal saline; (2) model group (M): airway instillation of lyophilized allergen powder suspension at 300 µL/20 µg once weekly; (3) M + D group: airway instillation of lyophilized allergen powder suspension at 300 µL/20 µg once weekly + tail vein injection of the Notch signaling pathway inhibitor dual-anti platelet-therapy (DAPT) at 0.05 mg/kg; (4) M + W group: airway instillation of lyophilized allergen powder suspension at 300 µL/20 µg once weekly + tail vein injection of the mitochondrial autophagy inhibitor wortmannin at 0.3 mg/kg; and (5) M + R group: airway instillation of lyophilized allergen powder suspension at 300 µL/20 µg once weekly + tail vein injection of the mitochondrial autophagy inducer rapamycin at 0.25 mg/kg.

During the modeling process, a total of four airway instillations of allergens were administered (once weekly in the first 4 weeks). DAPT and autophagy inducer or inhibitor were administered at the beginning of the model establishment, twice a week for 4 weeks; after the completion of the model establishment, they were administered once a week till the end of the experiment. The model was constructed using the above methods. At 20 weeks, pulmonary fibrosis was observed by hematoxylin and eosin (HE) and Masson staining. After the successful establishment of the model in the fibrotic phase, various indicators were observed and detected.

**Table 1:** Primary antibodies and their dilutions in this study

	Manufacturer	Dilution ratio
Primary antibody		
Notch1	Thermo Fisher	1:100
Notch2	Thermo Fisher	1:200
Jag1	Biorbyt	1:200
Jag2	Affinity	1:100
Dll1	Affinity	1:200
Dll4	Affinity	1:100
TGF-β1	ProteinTech	1:200
α-SMA	Abcam	1:200
LC3	ProteinTech	1:100
Beclin1	Abcam	1:200
Secondary antibody		
Anti-rabbit secondary antibody	Dako Denmark	1:200
Anti-mouse secondary antibody	Dako Denmark	1:200

#### 2.2.3 Observation indicators

(1) General conditions of rats. We mainly observed whether rats in each group had symptoms such as coughing, irritability, shortness of breath, and abdominal muscle twitching during the modeling process.
(2) Pathological changes in the lung tissues of rats in each group. The left lung tissue was placed in phosphate buffer containing 4% paraformaldehyde, and the specimens were fixed, dehydrated, paraffin-embedded, sectioned, deparaffinized, HE-stained, and Masson-stained. The morphological changes in pathological sections of lung tissue specimens were observed under an optical microscope (Nikon, H550S) (×200), and the degree of lung tissue fibrosis in each group was analyzed.
(3) Detection of the expression of α-SMA, TGF-β, Beclin1, LC3, and key proteins of the Notch signaling pathway (Notch1, Notch2, Jag1, Jag2, DLL1, and DLL4) in rat lung tissues by immunohistochemistry (IHC). Paraffin sections were baked in an oven at 65°C for 1 h, deparaffinized, hydrated to water, and rinsed with PBS three times for 5 min. The sections were retrieved in ethylenediaminetetraacetic acid antigen retrieval solution under high pressure for 10 min and then stopped retrieval. After natural cooling, the sections were washed with PBS three times. The sections were incubated with 3% hydrogen peroxide solution for 10 min at room temperature to block endogenous peroxidase. The sections were washed with PBS three times for 5 min each time and blocked with 5% bovine serum albumin (BSA) for 20 min (blocking charge) after spin-drying. The BSA solution was removed, and 100 μL of diluted primary antibody was added to cover the tissue, which was incubated at 4°C overnight. Sections were washed with PBS three times for 5 min, the PBS was removed, and 100 μL of secondary antibody of the corresponding species was added to each section. A horseradish peroxidase kit (MXB Biotechnology, Kit 5030, stock solution was added dropwise) was used to incubate the sections at 37°C for 30 min. Sections were washed with PBS three times for 5 min. After the PBS was removed, 100 μL of freshly prepared 3,3′-diaminobenzidine solution (MXB Biotechnology, DAB-1031, dilution ratio: 1:20) was added to each slide, and the color development was controlled under a microscope. After the color development was complete, the sections were rinsed with distilled water or tap water, counterstained with hematoxylin staining solution, differentiated with 1% hydrochloric acid alcohol (1 s), rinsed with tap water, blued in ammonia water, and rinsed with water. The sections were dehydrated with a gradient of ethanol (70–100%) for 10 min per concentration, dried, cleared with xylene, and sealed with neutral gum. The primary antibodies and their dilutions are given in Table 1.
(4) Detection of mitochondrial morphological changes in lung tissue using transmission electron microscopy. The experimental procedures were as follows: the tissues were fixed with 2.5% glutaraldehyde overnight at 4°C. They were rinsed three times with PBS for 10 min. They were fixed with 1% osmium tetroxide at room temperature for 1 h, then embedded with 10% gelatin. They were fixed with glutaraldehyde at 4°C for 1 h. They were dehydrated with increasing concentrations of ethanol solution (30, 50, 70, 90, 95, 100, 100, 100%). After the immersion and embedding with epoxy resin, the samples were sectioned by a Leica UC6 ultrathin microtome. Finally, under the 110 kV setting, the sample was observed and photographed using a transmission electron microscope (JEM-1011, Japan).
(5) Biochemical detection of ROS levels in serum and lung tissues of rats in each group. ROS levels in rat serum and lung tissue were detected using an ROS kit (Beitian, S0033), and the ROS content was calculated according to the kit manual. The experimental steps were as follows: (1) Blood specimens were collected from rats at the time of sacrifice, and the supernatant was collected after centrifugation and tested according to the instructions of the ROS assay kit. (2) Detection of ROS in lung tissue: lung tissue specimens were dissected and weighed, and the tissue was cut into pieces, homogenized in buffer, and filtered through 200 mesh to prepare the homogenate. The supernatant was collected for the assay after centrifugation for approximately 20 min (3,000 rpm). The specimens were tested as soon as possible after collection.

### 2.3 Statistical analysis

SPSS 21.0 software was used for statistical analysis of the obtained data. The measurement data are expressed as the mean ± standard deviation (*x* ± *s*). If the variance was homogeneous, the comparison between groups was performed by analysis of variance and by least significant difference multiple comparison; if the variance was not homogeneous, then a nonparametric test was used. *P* < 0.05 was considered statistically significant. GraphPad Prism v8 was used to draw graphs.

## 3 Results

### 3.1 Experimental results of lung pathology of rats in each group

#### 3.1.1 General conditions of rats

The rats in the control group were in good condition, with normal appetite and behavior. The rats in the model group and each intervention group gradually reduced their activities and were sluggish compared with the control group, with coughing, reduced appetite, no increase in body weight, and slower response to external stimuli.

#### 3.1.2 Pathological changes in rat lung tissue

**Figure 1:**
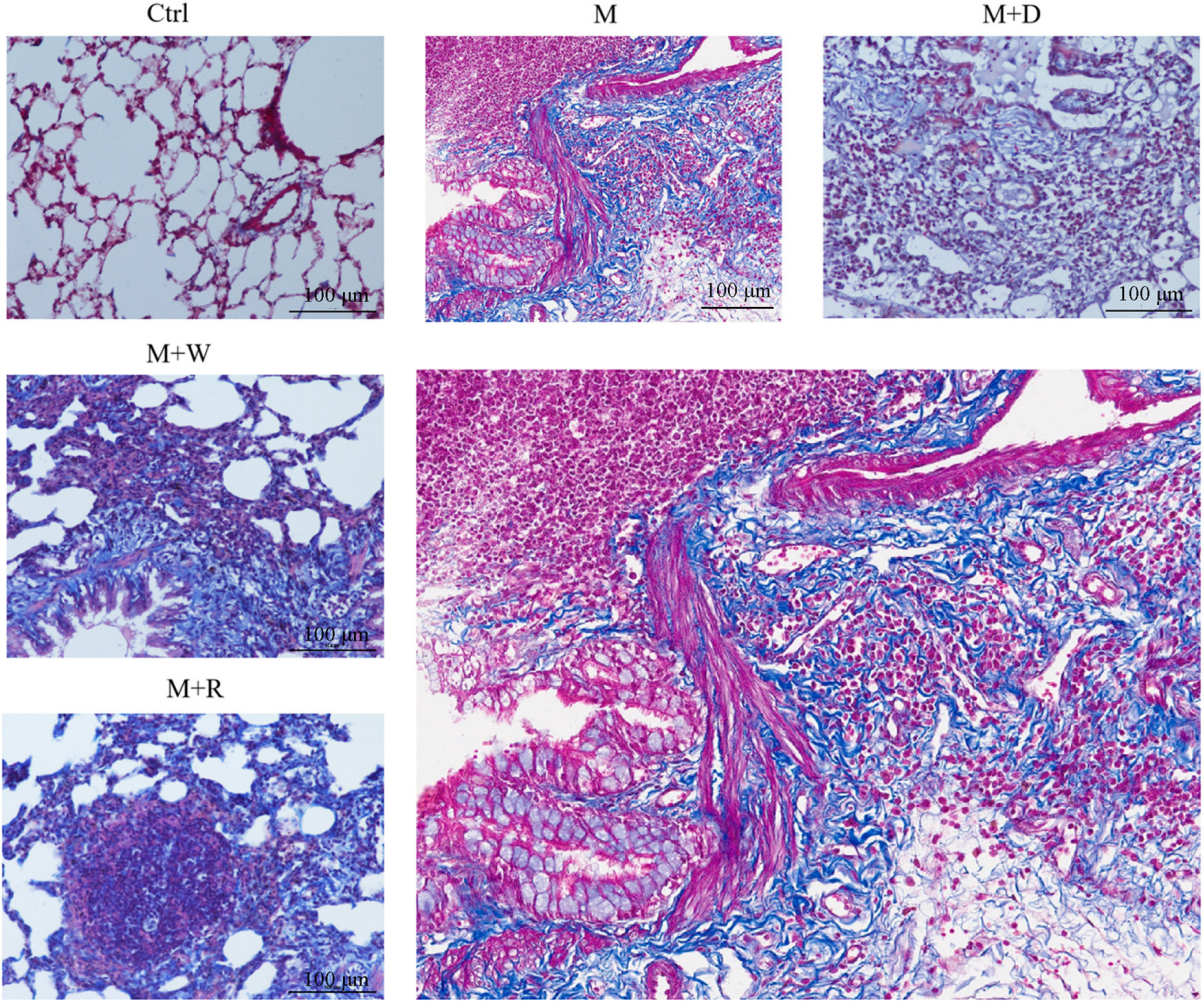
Masson staining pathological images of rat lung tissues in each group (Masson staining, 200×. The large image is the enlarged version of the group M image).

According to the results of the modeling in our pilot study, the rats were sacrificed on the 20th day of the modeling. The Masson staining results are shown in Figure 1. In the Ctrl group, there was no deposition of collagen fibers in the lung cells. In the rats of the M and M + W groups, there were more blue-stained collagen fibers in the alveoli, proliferation of collagen fibers in the wall of large blood vessels, collagen fibers around the inflammatory cells, and obvious interstitial fibrosis. Some alveolar septa were thickened, some alveolar structures disappeared, fibroblasts proliferated around the glands, and the fibrous tissues showed patchy extension. A small amount of collagen fiber deposition was observed in the M + D and M + R groups.

### 3.2 Expression levels of the pulmonary fibrosis marker α-SMA in rats of each group

**Figure 2:**
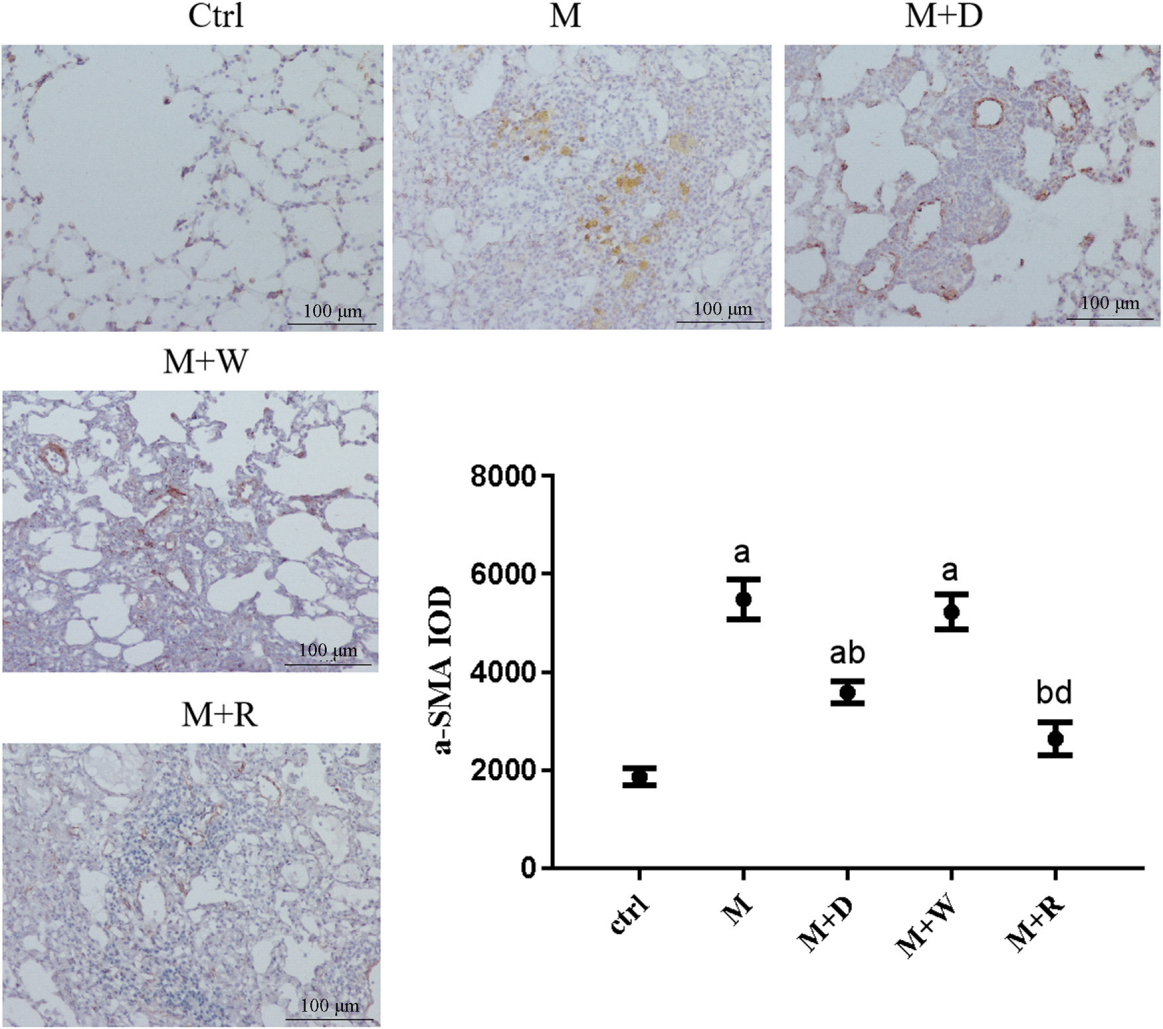
IHC image and expression levels of α-SMA, a fibrosis marker, in rat lung tissues of each group (×200): (a) statistically significant difference from the Ctrl group (*P* < 0.05), (b) statistically significant difference from the M group (*P* < 0.05), (c) statistically significant difference from the M + D group (*P* < 0.05), and (d) statistically significant difference from the M + R group (*P* < 0.05).

The expression of the pulmonary fibrosis marker α-SMA in the lung tissues of rats in each group was detected by IHC (Figure 2). The positive reaction signals of each key protein were brown–yellow particles, which were mainly located in the pulmonary interstitium. The more severe the inflammatory reaction, the stronger the positive reaction signal. The positive signal of the fibrosis marker α-SMA protein showed good consistency between the groups. Compared with that in the Ctrl group, the positive signal was stronger in the M and M + W groups, while the positive particles were significantly weaker in the M + D and M + R groups compared with the M group. Correspondingly, compared with that in the Ctrl group, the expression of α-SMA in the M group, the M + D group, and the M + W group was increased (*P* < 0.05). Compared with the M group, the M + D and M + R groups had lower α-SMA expression (*P* < 0.05). However, the expression of α-SMA was higher in the M + W group than the M + D and M + R groups (*P* < 0.05). The difference in the expression of α-SMA between the M + R group and Ctrl group was not statistically significant (*P* ≥ 0.05), nor was the difference between the M + W group and M group (*P* ≥ 0.05).

### 3.3 The relationship between the Notch signaling pathway in the lung tissues of rats and the occurrence of pulmonary fibrosis in PBL

**Figure 3:**
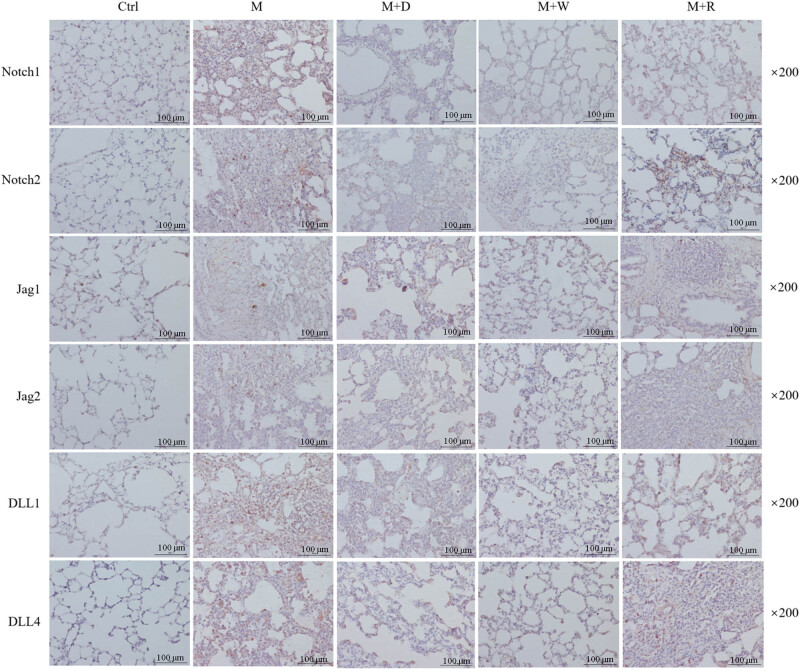
IHC image of key proteins of the Notch pathway in the lung tissues of rats in each group (×200).

**Figure 4:**
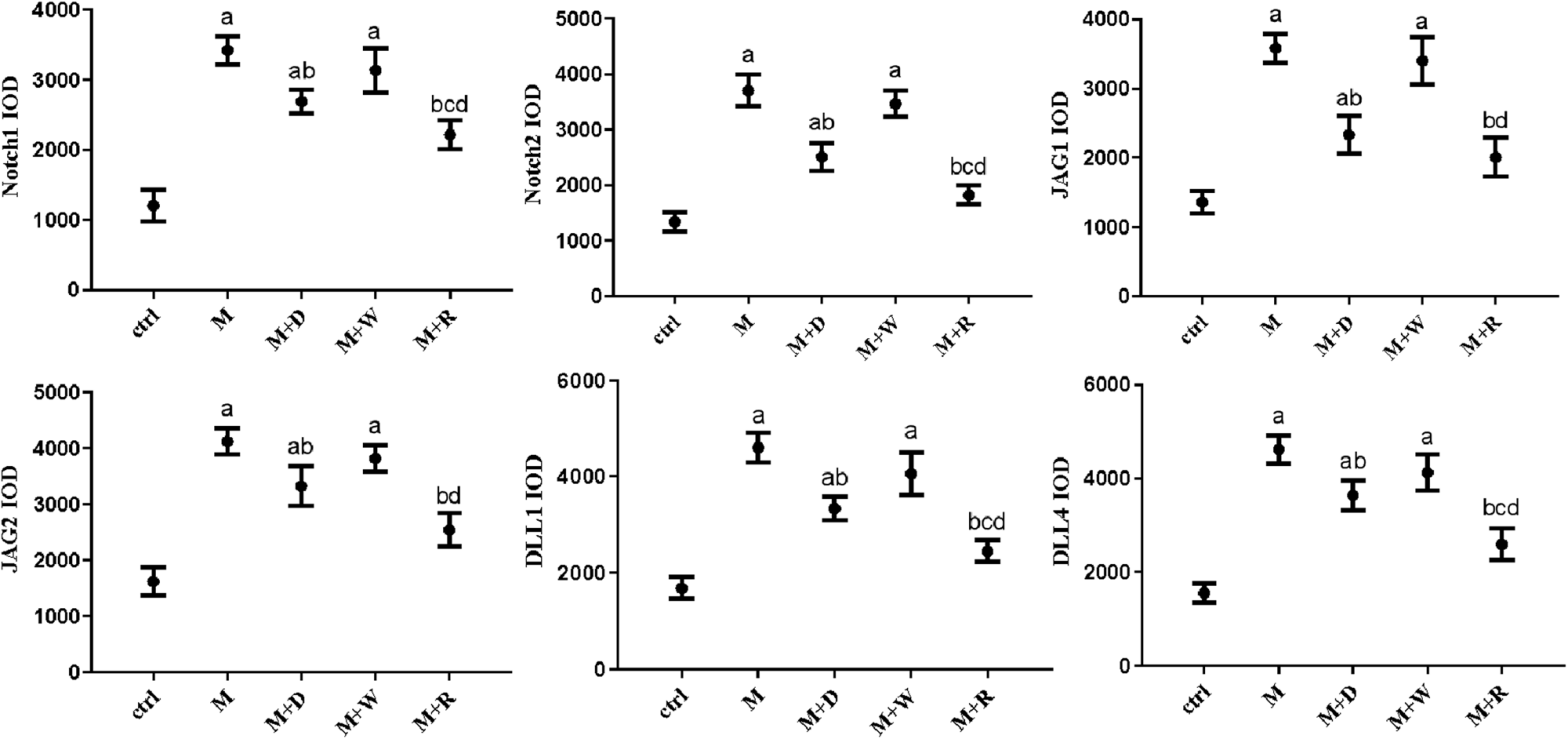
The expression levels of key proteins of the Notch pathway in the lung tissues of rats in each group: (a) statistically significant difference from the Ctrl group (*P* < 0.05), (b) statistically significant difference from the M group (*P* < 0.05), (c) statistically significant difference from the M + D group (*P* < 0.05), and (d) statistically significant difference from the M + R group (*P* < 0.05).

IHC analysis of the key proteins of the Notch pathway in the lung tissues of rats in each group was performed (Figures 3 and 4). Each group in the figures is represented in one column. Each row indicates the key proteins of Notch pathway that were detected: Notch1, Notch2, Jag1, Jag2, DLL1, and DLL4. The positive reaction signals of each key protein of the Notch signaling pathway were brown–yellow particles, which were mainly located in the lung interstitium. The positive reaction signal was stronger in the sites with a more severe inflammatory response. The positive signal of each key protein of the Notch signaling pathway showed good consistency between the groups. Compared with the Ctrl group, the M group and the M + W group had stronger signals, while the positive particles in the M + D group and the M + R group were significantly weaker than those in the M group. Correspondingly, the quantitative analysis of each key protein showed that the M group had higher expression than the Ctrl group (*P* < 0.05). The expression of each key protein in the M + D group and the M + R group was lower than that in the M group (*P* < 0.05). The expression of each key protein in the M + R group was lower than in the M + D group (*P* < 0.05), while the expression of each key protein in the M + W group was not significantly different from that in the M group (*P* ≥ 0.05).

**Figure 5:**
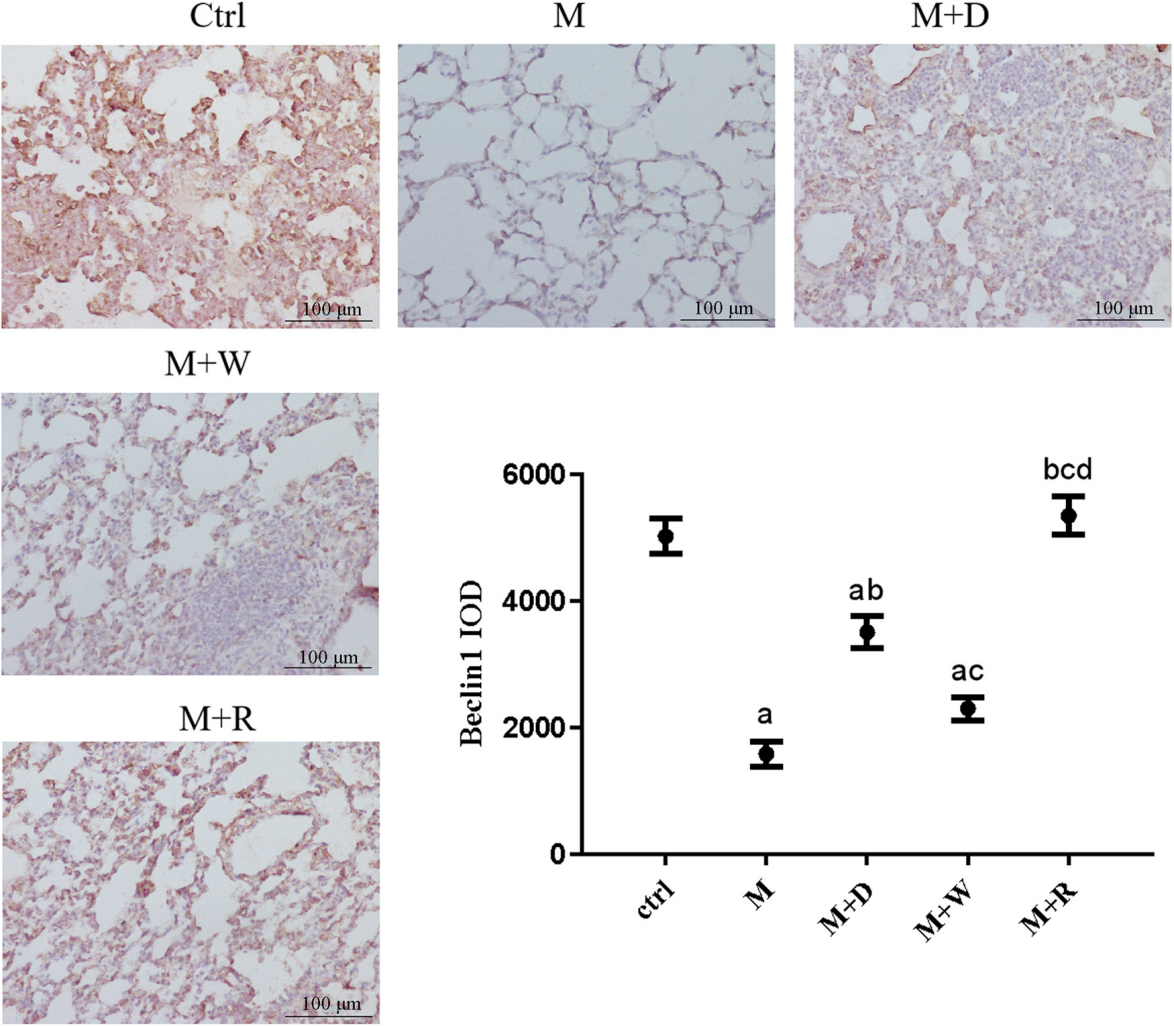
IHC image and expression levels of the mitochondrial autophagy marker Beclin1 in rat lung tissues of each group (×200): (a) statistically significant difference from the Ctrl group (*P* < 0.05), (b) statistically significant difference from the M group (*P* < 0.05), (c) statistically significant difference from the M + D group (*P* < 0.05), and (d) statistically significant difference from the M + W group (*P* < 0.05).

**Figure 6:**
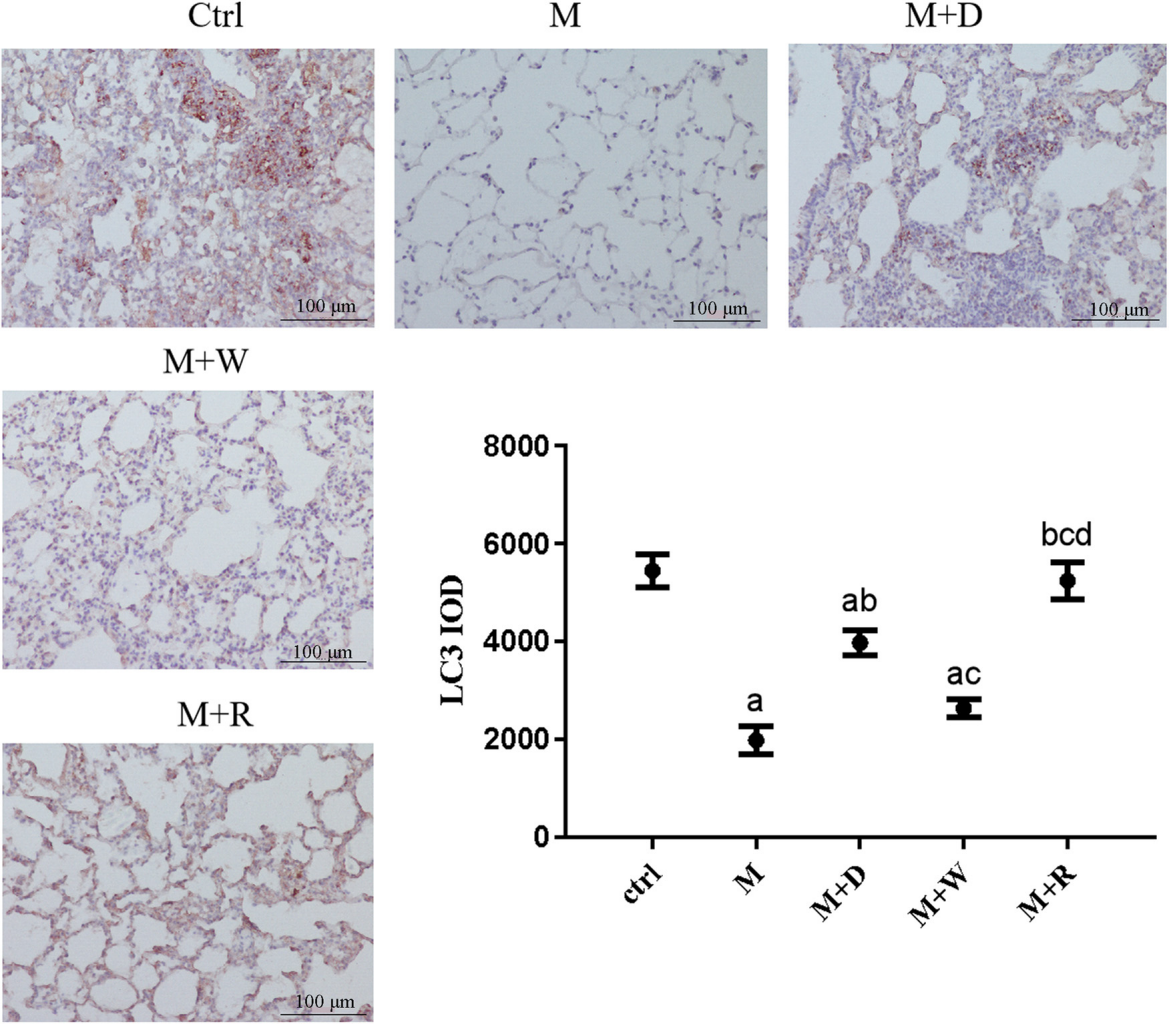
IHC image and expression levels of the mitochondrial autophagy marker Beclin1 in the lung tissues of rats in each group (×200): (a) statistically significant difference from the Ctrl group (*P* < 0.05), (b) statistically significant difference from the M group (*P* < 0.05), (c) statistically significant difference from the M + D group (*P* < 0.05), and (d) statistically significant difference from the M + W group (*P* < 0.05).

**Figure 7:**
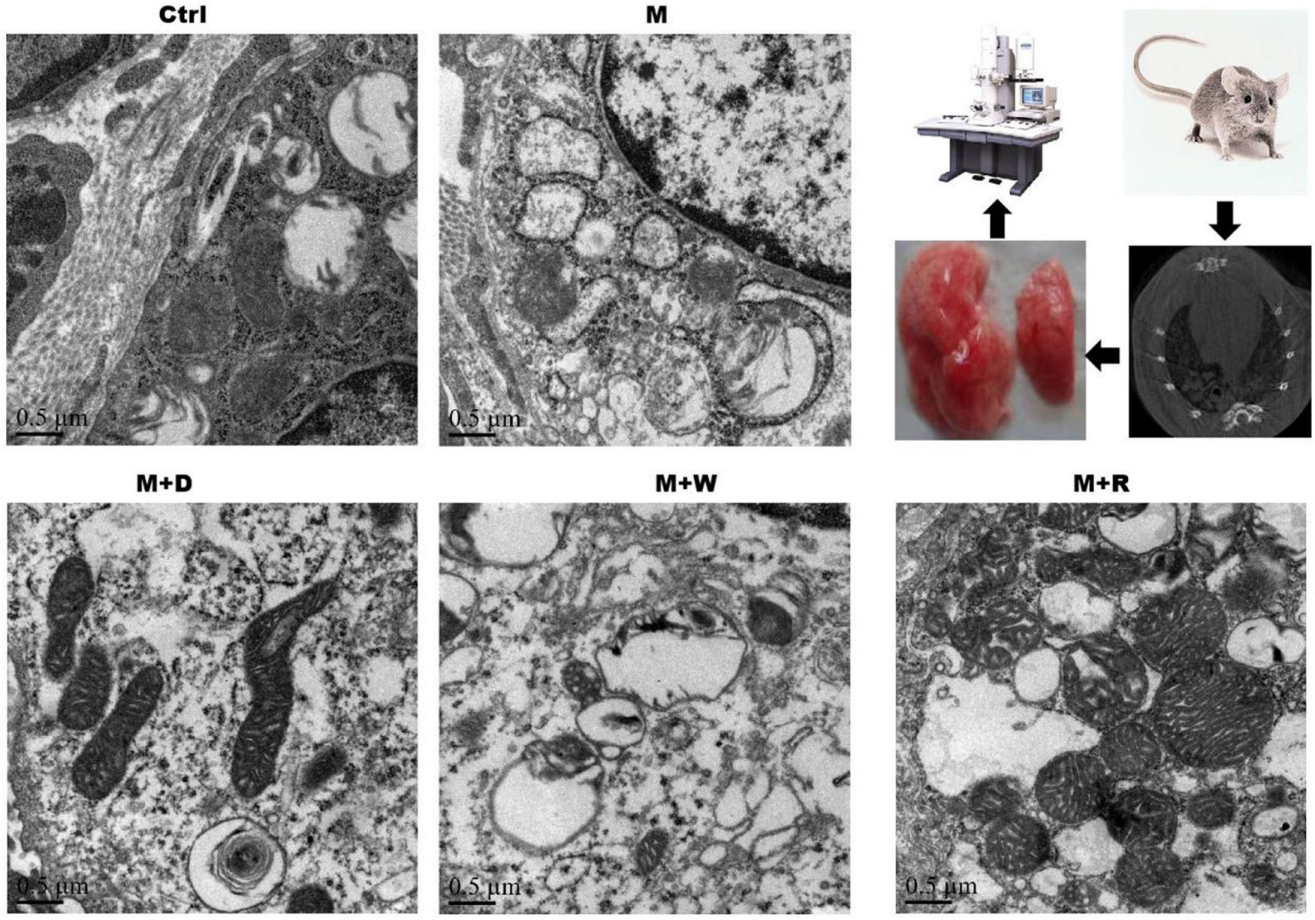
Electron microscopy of the mitochondrial structure of the lung tissue in each group. The observation flow chart is shown in the upper right panel.

**Figure 8:**
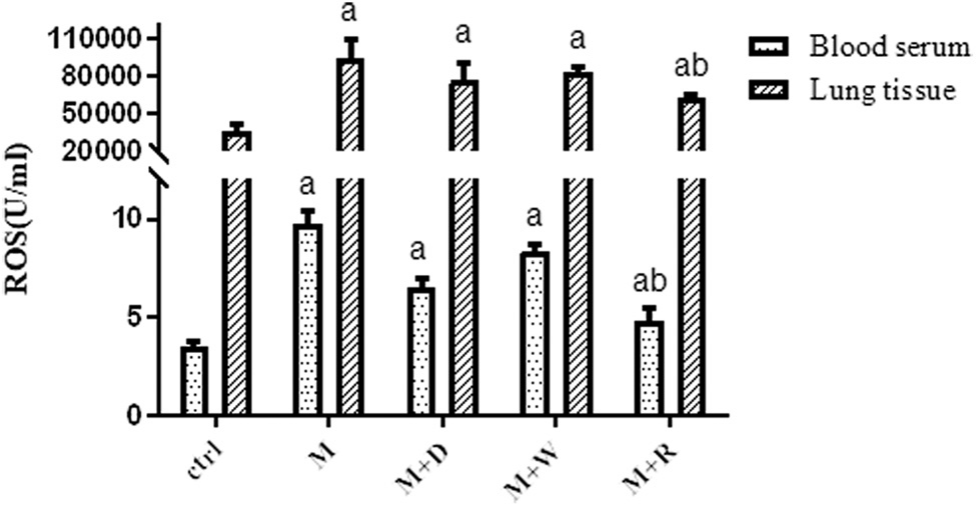
Analysis of the expression levels of oxidative stress products in the serum and lung tissues of rats in each group: (a) statistically significant difference from the Ctrl group (*P* < 0.05) and (b) statistically significant difference from the M group (*P* < 0.05).

### 3.4 Expression of the mitochondrial autophagy markers Beclin1 and LC3 proteins in rat lung tissues and the results of transmission electron microscopy

(1) Two key mitochondrial autophagy proteins, Beclin1 and LC3, were detected in lung tissues (Figures 5 and 6). The positive reaction signals of each key protein were brown–yellow particles, which were mainly located in the lung interstitium. The positive reaction signal was stronger in the site with a more severe inflammatory response. Positive signals of each key protein of mitochondrial autophagy showed good consistency between the groups. Compared with the Ctrl group, the positive signal was stronger in the M and M + W groups, while the positive particles were significantly weaker in the M + D and M + R groups than the M group. Correspondingly, the quantitative analysis of each key protein showed that Beclin1 and LC3 expression were lower in the M group, the M + D group, and the M + W group than in the Ctrl group (*P* < 0.05). Beclin1 and LC3 expression were higher in the M + D and M + R groups than in the M group (*P* < 0.05), and Beclin1 and LC3 expression were lower in the M + W group than in the M + D and M + R groups (*P* < 0.05). The differences of the expression of Beclin1 and LC3 between the M + R group and the Ctrl group were not statistically significant (*P* ≥ 0.05), nor were they between the M + W group and the M group (*P* ≥ 0.05).
(2) The mitochondrial structure of the lung tissues of each group under the electron microscope is shown in Figure 7. The mitochondrial structure of the lung tissues of the Ctrl group was normal. The lung mitochondria of the M group and the M + W group were swollen, their cristae were broken or missing, and the lamina was smaller, accompanied by local focal lysis. The swelling of mitochondria in the M + D and M + R groups was significantly relieved or restored to normal, and they had significantly more lamellar bodies.

### 3.5 The involvement of the Notch signaling pathway and mitochondrial autophagy in pulmonary fibrosis-related oxidative stress in PBL

The levels of ROS in serum and lung tissues were also measured (Figure 8). Compared with the Ctrl level, the serum ROS levels in the M group, the M + D group, the M + R group, and the M + W group were increased (*P* < 0.05). The serum ROS level in the M + R group was lower than that of the M group (*P* < 0.05).

## 4 Discussion

**Figure 9:**
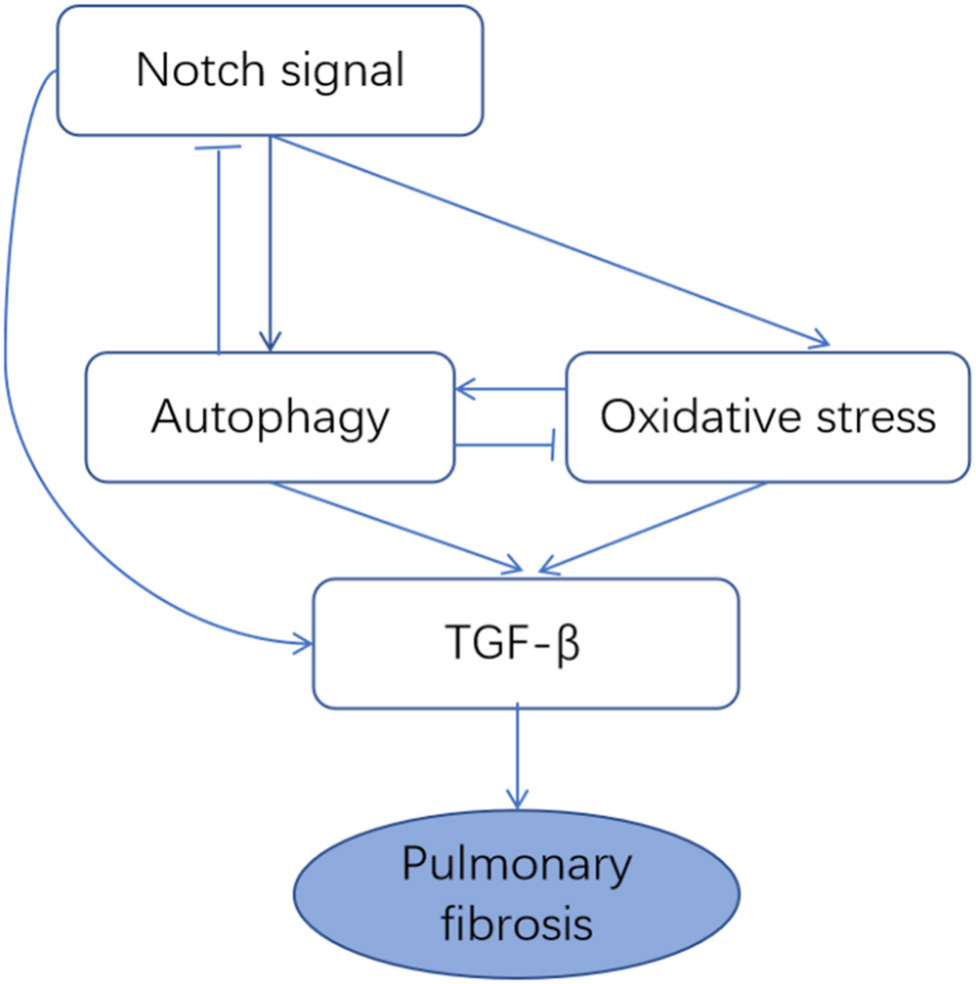
Hypothesis for the mechanism of Notch signaling pathway-mediated autophagy involvement in pulmonary fibrosis.

Pulmonary fibrosis is closely related to Notch signaling pathway abnormalities, cell autophagy, and oxidative stress. However, the mechanism underlying their interaction and its effect on pulmonary fibrosis in HP must be further studied. This study found that Notch signaling pathway-mediated alterations in autophagy were involved in the pathological process of pulmonary fibrosis in a rat model of PBL. Inhibition of the Notch pathway and intervention of autophagy may be among the mechanisms for slowing the progression of pulmonary fibrosis. The hypothesized mechanism of the involvement of Notch pathway-mediated autophagy in pulmonary fibrosis in PBL is shown in Figure 9.

In this study, lyophilized protein powder from pigeon shedding materials was used as an allergen to construct a fibrotic PBL rat model by airway instillation. According to our previous experiment [15], Masson staining of lung tissues of rats in the 20th week of modeling demonstrated that all experimental groups showed fibrosis, with destruction of the alveolar structure and deposition of collagen fibers, but the degree of fibrosis was different between the groups. The Notch signaling pathway inhibition group had less fibrosis than the model group. The detection of α-SMA, a marker of lung fibrosis, in each group showed that the expression of α-SMA increased after the occurrence of lung fibrosis. Expression of α-SMA decreased in the Notch signaling pathway intervention group compared with the model group, and the expression of α-SMA was consistent with the degree of lung tissue fibrosis. At the same time, the detection of the Notch signaling pathway in the lung tissues of rats in each group showed that the expression of key proteins in the Notch signaling pathway in the experimental group was higher than that in the control group (*P* < 0.05), while the expression of key proteins in the Notch signaling pathway blockade group was decreased. The above results suggest that there was abnormal activation of the Notch signaling pathway during PBL fibrosis. Inhibition of the Notch signaling pathway reduced the degree of fibrosis, which is consistent with studies on bleomycin-induced pulmonary fibrosis [16]. Studies on renal fibrosis and liver fibrosis also suggest that inhibition of the Notch signaling pathway can reduce the degree of organ fibrosis [17,18]. γ-Secretase inhibitors (DAPTs) are a class of inhibitors targeting the Notch signaling pathway. The inhibition of Notch signaling by DAPT can inhibit the transformation of fibroblasts into myofibroblasts, an important link in pulmonary fibrosis, which significantly reduces the expression of α-SMA, thereby reducing the generation and deposition of extracellular matrix [19]. Therefore, DAPT has a certain effect on inhibiting the progression of pulmonary fibrosis in PBL.

Autophagy is closely related to pulmonary fibrosis. Beclin1 and LC3 are key protein molecules in the process of autophagy, and their changes can represent the level of autophagy in cells [20]. To further study the mechanism underlying the involvement of the Notch pathway and cell autophagy in the pulmonary fibrosis of PBL, these two key proteins in mitochondrial autophagy in lung tissues were detected, and the results suggested decreased levels of autophagy after the development of pulmonary fibrosis. In the Notch pathway inhibition group and the autophagy induction group, Beclin1 and LC3 expression were increased, and the degree of pulmonary fibrosis was alleviated. The expression of key proteins of the Notch pathway was reduced in the autophagy induction group. Transmission electron microscopy showed that in the Notch inhibition and autophagy induction groups, mitochondrial enlargement in lung tissue decreased significantly, and the number of normal lamellar bodies significantly increased. The above results suggest that induction of autophagy and inhibition of Notch signaling can mitigate pulmonary fibrosis, while inhibition of the Notch pathway upregulates autophagy-related proteins. Reduced expression of Notch pathway receptors and ligands after induction of autophagy suggests that inhibition of the Notch pathway may relieve lung fibrosis by improving the level of cellular autophagy, while inhibition of autophagy may be involved in the pulmonary fibrosis process by affecting the Notch signaling pathway. The loss of mitochondrial lysis after inhibition of autophagy is thought to be related to the abnormal mitochondrial function caused by autophagy dysfunction, i.e., the Notch pathway and autophagy are mutually regulated and participate in the pulmonary fibrosis process of HP, but the mechanism of their interaction is still unclear. Studies related to bleomycin-induced pulmonary fibrosis in rats have also shown impaired autophagic activity in idiopathic pulmonary fibrosis [14,21], and inhibition of autophagy affected the pulmonary fibrosis process, in line with the results of this study. Studies on polycystic ovary syndrome also suggest that the Notch signaling pathway has a regulatory effect on autophagy [22].

The mammalian target of rapamycin (mTOR) signaling pathway is an important negative regulator of autophagy [23]. mTOR is inhibited by rapamycin, and autophagy is regulated and activated by the Akt/mTOR signaling pathway to reduce the occurrence and development of fibrosis, which is consistent with the conclusions of a study of renal tubular fibrosis [24]. There is crosstalk between autophagy and the Notch signaling pathway [25–28]. These two signaling pathways are intertwined with each other and can be simultaneously affected by other mechanisms to promote cell–cell interactions. In pulmonary fibrosis, TGF-β is the most important EMT initiation factor. Notch signaling can induce EMT in alveolar epithelial cells through TGF-β1, and then pulmonary interstitial fibrosis occurs. At the same time, TGF-β1 activates autophagy, but the two have opposite mechanisms of action in pulmonary fibrosis. The ROS produced during oxidative stress can induce autophagy, and autophagy can alleviate the damage caused by oxidative stress, thereby protecting cell survival. Autophagy can delay the pulmonary fibrosis process by inhibiting the production of ROS [10].

ROS play a role in enhancing TGF-β signaling and promoting fibrosis signal transduction. ROS are a normal product of redox reactions in the body. When excessive accumulation of ROS cannot be removed, it can induce oxidative stress damage and lead to lung tissue damage and remodeling, thereby promoting pulmonary fibrosis [11]. To study autophagy and oxidative stress after the pulmonary fibrosis, our detection of ROS in serum and lung tissues suggested that ROS increased after the pulmonary fibrosis, while the ROS content was lower than in the model group after induction of autophagy. The above findings suggest that the expression of the Notch pathway was upregulated, the level of autophagy decreased, and there was accumulation of ROS after the occurrence of pulmonary fibrosis in PBL. The decrease in Notch signaling pathway component expression, ROS levels, and lung fibrosis after induction of autophagy may be related to the decrease in lung fibrosis due to the lesser oxidative stress damage, thanks to increased scavenging of ROS after induction of autophagy. A reduction in ROS reduces the release of TGF-β by weakening its stimulation [29], while a reduction in Notch activation together reduces EMT and thus alleviates pulmonary fibrosis [29]. The lower fibrosis may also be related to the protective effect that Notch signaling has on pulmonary fibrosis by reducing oxidative stress damage, which is consistent with the finding in that curcumin protects intravascular cells from damage caused by oxidative stress [30]. Oxidative stress contributes to the induction and persistence of TGF-1-induced pulmonary fibrosis [31]. Other studies showed that after LC3-B and Beclin-1 gene silencing, the expression of myofibroblast markers such as α-SMA and fibronectin significantly increased, indicating that inhibition of autophagy activation could promote myofibroblast proliferation and extracellular matrix deposition [32]. This study also suggested that the degree of pulmonary fibrosis increased after the inhibition of autophagy, which may be related to the above factors. However, excessive autophagy is also a kind of damage to cells [33]. The difference in autophagy levels in PBL at different pathological stages remains to be further studied.

This study has some limitations. First, this is an animal experiment, and there is a large difference between the airway instillation method and the actual pathogenesis. The onset time of the human disease after exposure to pigeon antigens is usually several years to several decades, but the disease onset of the PBL animal model is much shorter. Second, the number of animals used in this experiment was small, which may have had an impact on the accuracy of the experimental results. This study preliminarily analyzed the role of the mutual regulation between the Notch signaling pathway and cell autophagy in the development of pulmonary fibrosis. The cells involved in the pathogenesis of this process could be further investigated by using a cytology model of PBL. Animal model experiments are always only indirect research methods on human diseases, and the correctness of their conclusions must be verified in humans.

In summary, this study confirms that Notch signaling and autophagy play important roles in the development of pulmonary fibrosis in a rat model of HP. We further revealed the effects of inhibiting autophagy and Notch signaling on pulmonary fibrosis. We elucidated the significance of the mutual regulation of Notch signaling pathway and autophagy in the development of pulmonary fibrosis. This study provides an important molecular target and theoretical basis for the treatment and prevention of HP in clinical practice.
